# The Peroxisome Proliferator-Activated Receptor (PPAR) α Agonist Fenofibrate Suppresses Chemically Induced Lung Alveolar Proliferative Lesions in Male Obese Hyperlipidemic Mice

**DOI:** 10.3390/ijms15059160

**Published:** 2014-05-22

**Authors:** Toshiya Kuno, Kazuya Hata, Manabu Takamatsu, Akira Hara, Yoshinobu Hirose, Satoru Takahashi, Katsumi Imaida, Takuji Tanaka

**Affiliations:** 1 Department of Experimental Pathology and Tumor Biology, Graduate School of Medical Sciences, Nagoya City University, Nagoya 467-8601, Japan; E-Mail: sattak@med.nagoya-cu.ac.jp; 2 Department of Tumor Pathology, Graduate School of Medicine, Gifu University, Gifu 501-1194, Japan; E-Mails: k-hata-sun@hhc.eisai.co.jp (K.H.); sw20.3s-gte@hotmail.co.jp (M.T.); ahara@gifu-u.ac.jp (A.H.); takutt@toukaisaibou.co.jp (T.T.); 3 Department of Pathology, Osaka Medical College, Osaka 569-8686, Japan; E-Mail: hirose@art.osaka-med.ac.jp; 4 Onco-Pathology, Department of Pathology and Host-Defenses, Faculty of Medicine, Kagawa University, Kagawa 761-0793, Japan; E-Mail: imaida@med.kagawa-u.ac.jp; 5 Department of Diagnostic Pathology & Research Center of Diagnostic Pathology, Gifu Municipal Hospital, Gifu 500-8513, Japan

**Keywords:** 4-nitroquinoline-1-oxide, lung neoplasms, carcinogenesis, hyperlipidemia, hyperinsulinemia, chemoprevention

## Abstract

Activation of peroxisome proliferator-activated receptor (PPAR) α disrupts growth-related activities in a variety of human cancers. This study was designed to determine whether fenofibrate, a PPARα agonist, can suppress 4-nitroquinoline 1-oxide (4-NQO)-induced proliferative lesions in the lung of obese hyperlipidemic mice. Male Tsumura Suzuki Obese Diabetic mice were subcutaneously injected with 4-NQO to induce lung proliferative lesions, including adenocarcinomas. They were then fed a diet containing 0.01% or 0.05% fenofibrate for 29 weeks, starting 1 week after 4-NQO administration. At week 30, the incidence and multiplicity (number of lesions/mouse) of pulmonary proliferative lesions were lower in mice treated with 4-NQO and both doses of fenofibrate compared with those in mice treated with 4-NQO alone. The incidence and multiplicity of lesions were significantly lower in mice treated with 4-NQO and 0.05% fenofibrate compared with those in mice treated with 4-NQO alone (*p* < 0.05). Both doses of fenofibrate significantly reduced the proliferative activity of the lesions in 4-NQO-treated mice (*p* < 0.05). Fenofibrate also significantly reduced the serum insulin and insulin-like growth factor (IGF)-1 levels, and decreased the immunohistochemical expression of IGF-1 receptor (IGF-1R), phosphorylated Akt, and phosphorylated Erk1/2 in lung adenocarcinomas. Our results indicate that fenofibrate can prevent the development of 4-NQO-induced proliferative lesions in the lung by modulating the insulin-IGF axis.

## 1. Introduction

Despite advances in the medical treatment of lung cancer, it is still a leading cause of cancer-related death in men and women in the United States and men in Japan [1,2]. Smoking is the major cause of lung cancer. Although smoking cessation can reduce lung cancer mortality, former smokers have a life-long higher risk of lung cancer compared with never-smokers. In recent years, a number of molecular targeted therapies, such as epidermal growth factor receptor (EGFR) tyrosine kinase inhibitors and an inhibitor of echinoderm microtubule-associated protein-like 4-anaplastic lymphoma receptor tyrosine kinase (EML4-ALK), have been developed for the treatment of non-small cell lung cancer. Despite the success of genotype-directed therapies in EGFR-mutant and ALK-positive patients, resistance necessarily develops. The median progression free survival after treatment with EGFR or ALK inhibitors in target populations is generally less than 1 year [3]. Therefore, prevention is the most effective tools of decreasing lung cancer development [4].

Recent epidemiological studies have shown that diabetes mellitus and dyslipidemia may increase the risk of lung cancer [5,6]. Although the precise mechanisms by which these chronic diseases promote pulmonary carcinogenesis are still unknown, insulin resistance and hyperinsulinemia are thought to be responsible for the increased risk of developing lung cancer [7]. Insulin resistance, hyperinsulinemia, and hyperglycemia caused by excess body weight are thought to be involved in tumor development in several tissues, including the lung [8]. A preclinical study revealed that metformin, an antidiabetic drug that improves insulin resistance, inhibited lung carcinogenesis [9]. Furthermore, metformin is associated with a lower risks of colorectal cancer, hepatocellular cancer, and lung cancer [10], and thus the chemopreventive and therapeutic potential of metformin in non-small cell lung cancer is currently being evaluated in several clinical trials, some of which have advanced to Phase III (e.g., NCT01717482, NCT01997775).

The insulin-like growth factor 1 receptor (IGF-1R) is a tyrosine kinase receptor that belongs to a family of two ligands (IGF-1 and IGF-2), three receptors (IGF-1R, IGF-2R, and insulin receptor), and seven binding proteins (IGFBP1–7). Activation of IGF-1R is implicated in carcinogenesis, and the IGF receptor pathway is a molecular target of lung cancer therapy [11]. Murine lung tumors induced by a potent tobacco smoke carcinogen, 4-(methylnitrosamino)-1-(3-pyridyl)-1-butanone (NNK), expressed high levels endogenous IGF-1R protein, which regulates the phosphatidyl inositide 3-kinase (PI3K)/Akt and mitogen-activated protein kinase (MAPK)/extracellular signal-related kinase (ERK) pathways [12]. In addition, insulin can enhance IGF signaling by directly stimulating IGF-1R [13]. A recent report has suggested that IGF signaling may play an important role in the early stage of non-small cell lung cancer patients with diabetes [14].

Fenofibrate, a peroxisome proliferator-activated receptor (PPAR) α ligand, is an effective hypolipidemic agent [15]. Fenofibrate also had beneficial effects on atherogenic dyslipidemia in patients with metabolic syndrome or type 2 diabetes mellitus by reducing triglyceride (TG) levels, tending to increase high-density lipoprotein–cholesterol (HDL-C) levels, and promoting a shift to larger low-density lipoprotein particles [16]. It was recently reported that fenofibrate inhibited IGF-1 mediated growth of a medulloblastoma cell line [17]. Moreover, activation of PPARα with fenofibrate inhibited the IGF-1R signaling pathway [17]. These effects of fenofibrate have been demonstrated in a variety of cancer cell lines established from rodents and humans [18,19]. However, very few *in vivo* studies have investigated the effects of fenofibrate on carcinogenesis. We recently reported that bezafibrate, another PPARα agonist, inhibited carcinogenesis in the mouse colon [20]. Since fenofibrate has relatively low systemic toxicity [21] and has cardioprotective properties [22], its potential chemopreventive effects in obesity- and metabolic syndrome-related carcinogenesis is an attractive proposition.

Tsumura Suzuki Obese Diabetic (TSOD) mice, which were established from the ddY strain [23], rapidly accumulate visceral fat from 4 weeks of age and show similar symptoms, such as insulin resistance, to those observed in people with metabolic syndrome. 4-Nitroquinoline 1-oxide (4-NQO) is a pluripotent carcinogen in several tissues, and is frequently used to induce oral [24] and lung [25,26] cancers *in vivo*. 4-NQO was reported to induce the development of lung adenoma and adenocarcinoma in ddY mice [25]. Because the effects of fenofibrate on lung carcinogenesis have not been studied, the present study examined whether dietary fenofibrate can suppress 4-NQO-induced lung tumorigenesis in male TSOD mice. We also determined the modulatory effects of fenofibrate on plasma IGF-1 levels and immunohistochemical expression of IGF-1R, phosphorylated (p)-Akt, p-Erk1/2, PPARα and PPARγ in lung lesions induced by 4-NQO.

## 2. Results and Discussion

### 2.1. Results

#### 2.1.1. General Observation

All animals remained healthy throughout the experimental period. The mean body, liver, and relative liver weights in all groups at the end of the study are listed in Table S1. The mean body weights of mice in groups 2 (4-NQO—0.01% fenofibrate, *p* < 0.001) and 3 (4-NQO—0.05% fenofibrate, *p* < 0.001) were significantly lower than that of group 1 (4-NQO alone). The mean relative liver weight of group 3 (*p* < 0.001) was significantly greater than that of group 1, while the relative liver weight of group 4 (0.05% fenofibrate alone) was significantly greater than that of group 5 (control group, *p* < 0.001).

#### 2.1.2. Incidence and Multiplicity of Pulmonary Proliferative Lesions

**Figure 1.**
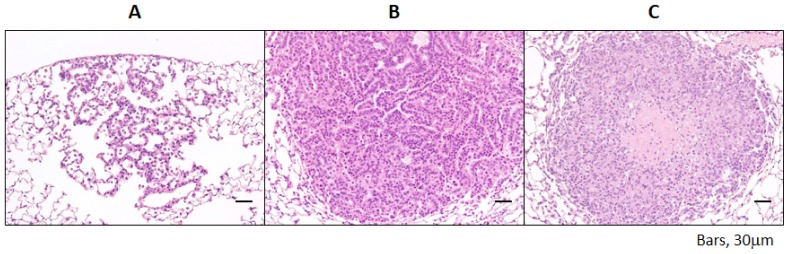
Histopathologic features of representative pulmonary proliferative lesions (hematoxylin and eosin stain). (**A**) bronchioloalveolar hyperplasia; (**B**) adenoma; (**C**) adenocarcinoma. There are numerous hyperplastic alveolar cells with an alveolar structure in bronchioloalveolar hyperplasia while tumor cells without an alveolar structure are present in adenoma. The central area of the adenocarcinoma is necrotic.

Administration of 4-NQO induced the development of lung proliferative lesions (Figure 1A–C) that were histopathologically diagnosed as bronchioloalveolar hyperplasia (Figure 1A); adenoma (Figure 1B); and adenocarcinoma (Figure 1C).

As summarized in Table 1, adenomas developed in all five groups, including the untreated group, but only one mouse in each of groups 3 (incidence: 4%), 4 (incidence: 8%), and 5 (incidence: 8%) had an adenoma. 4-NQO induced the development of all types of lung-proliferating lesions, including adenocarcinoma (Table 1). Mice in group 2 developed alveolar hyperplasia and adenoma, while those in group 3 only developed adenoma. When all of the types of proliferative lesions were combined, the incidence (4%, *p* < 0.05) and multiplicity (0.04 ± 0.20, *p* < 0.05) were significantly lower in group 3 than in group 1 (incidence: 33%; multiplicity: 0.46 ± 0.78).

#### 2.1.3. Immunohistochemical Expression of IGF-1R, p-Akt, p-Erk1/2, PPARα, and PPARγ in Pulmonary Proliferative Lesions Induced by 4-NQO

**Figure 2.**
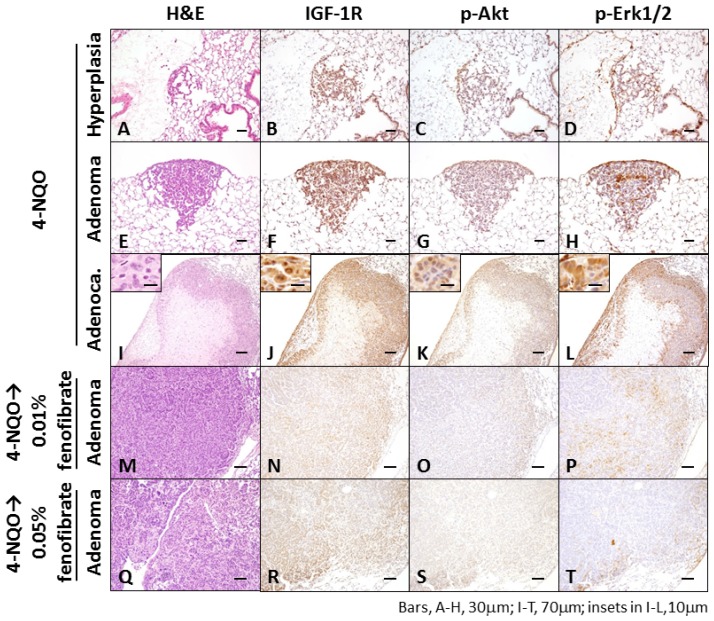
Histopathology (**A**,**E**,**I**,**M**,**Q**) and immunohistochemistry (insulin-like growth factor 1 receptor (IGF-1R), phosphorylated (p)-Akt, and p-extracellular signal-related kinase (Erk) 1/2) of bronchioloalveolar hyperplasia (**B**–**D**,**N**,**O**,**P**,**R**–**T**), adenoma (**F**–**H**), and adenocarcinoma (**J**–**L**) induced by 4-nitroquinoline 1-oxide (4-NQO) administration in Tsumura Suzuki Obese Diabetic (TSOD) mice. The adenomas **M**–**P** and **Q**–**T** are developed in a mouse treated with 4-NQO followed by dietary exposure to 0.01% and 0.05% fenofibrate, respectively. Alveolar hyperplasia (**A**–**D**), adenoma (**E**–**H**), and adenocarcinoma (**I**–**L**) were positively stained with IGF-1R (**B**,**F**,**J**), p-Akt (**C**,**G**,**K**), and p-Erk1/2 (**D**,**H**,**L**) antibodies, respectively. Note the weakly positive or negative reactions against IGF-1R, p-Akt, and p-Erk1/2 of adenoma in mice that received 4-NQO and fenofibrate. (**A**,**E**,**I**,**M**,**Q**): H&E stain; (**B**,**F**,**J**,**N**,**R**): IGF-1R immunohistochemistry; (**C**,**G**,**K**,**O**,**S**): p-Akt immunohistochemistry; and (**D**,**H**,**L**,**P**,**T**): p-Erk1/2 immunohistochemistry.

The immunohistochemical expression levels of IGF-1R, p-Akt, and p-Erk1/2 were examined in 4-NQO-induced pulmonary proliferative lesions (Figure 2). 4-NQO induced the expression of IGF-1R, p-Akt, and p-Erk1/2 in all proliferative lesions, and the expression levels of IGF-1R and p-Erk1/2 were greater than the levels of p-Akt in each lesion, as shown in Figure 2. Expression of IGF-1R, p-Akt, and p-Erk1/2 was weak or lacking in the adenomas by the treatment with fenofibrate.

**Figure 3.**
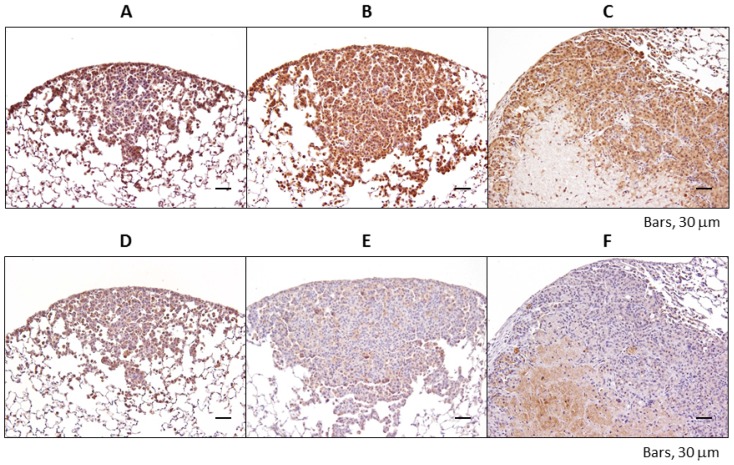
Immunohistochemical staining of PPARα (**A**–**C**) and PPARγ (**D**–**F**) in TSOD mice treated with 4-NQO. Representative slides show a strong increase in PPARα expression in the nuclei of lung proliferative lesions: (**A**,**D**): bronchioloalveolar hyperplasia; (**B**,**E**): adenoma; and (**C**,**F**): adenocarcinoma.

Regarding PPARα (Figure 3A–C), its expression was upregulated in the cytoplasm and nucleus of 4-NQO-induced lung lesions as the lesions progressed from bronchioloalveolar hyperplasia to adenocarcinoma. Although PPARγ protein expression was increased in the nucleus of hyperplasic lesions, it was not expressed in the nuclei of adenoma or adenocarcinoma (Figure 3D–F).

#### 2.1.4. Ki-67 Labeling Index of Pulmonary Proliferative Lesions

**Figure 4.**
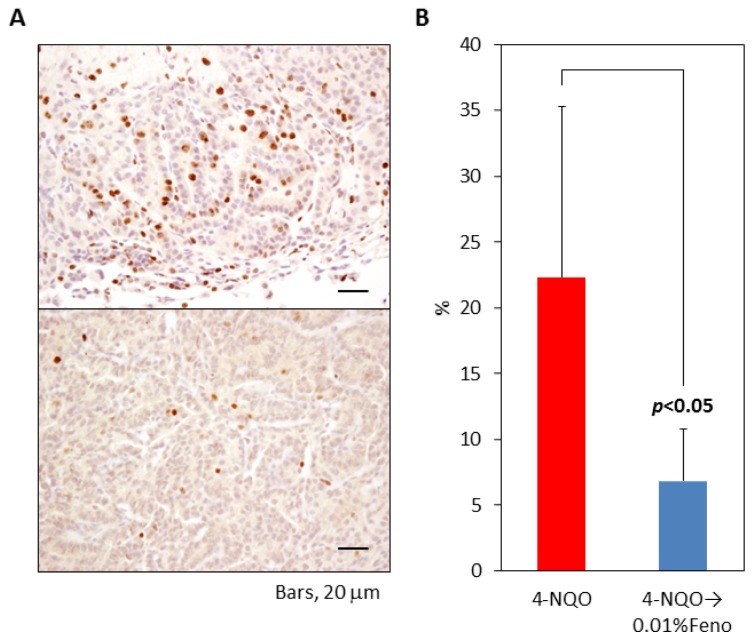
Ki-67 labeling index and immunohistochemistry of 4-NQO-induced pulmonary proliferative lesions from mice treated with or without fenofibrate. (**A**) Representative features of Ki-67 immunohistochemistry in a mouse treated with 4-NQO alone (upper; group 1) or 4-NQO followed by 0.01% fenofibrate (lower; group 2). Bars = 20 μm; (**B**) Dietary administration of 0.01% fenofibrate significantly lowered the Ki-67 labeling index in group 2 compared with mice treated with 4-NQO alone (group 1) (*p* < 0.05).

The proliferative potential of pulmonary proliferative lesions was estimated as the Ki-67 labeling index, as illustrated in Figure 4. The Ki-67 labeling index (6.8%) of pulmonary proliferative lesions was significantly lower in group 2 than in group 1 (22.3%, *p* < 0.05). The Ki-67 labeling index was 10.9% and 5.4% in an adenoma that developed in a mouse in groups 3 and 4, respectively.

#### 2.1.5. Serum Triglyceride, Free Fatty Acid, Total Cholesterol, Glucose, Insulin, and Insulin-Like Growth Factor-1 Levels

The serum triglyceride (TG), free fatty acid (FFA), total cholesterol (TC), glucose, insulin, and IGF-1 levels of 36-week-old mice are listed in Table 2. Dietary feeding with fenofibrate at a dose of 0.05% significantly lowered the levels of TG, FFA, and insulin. TC level showed a slight decrease in the fenofibrate-treated groups when compared to that given the diets without fenofibrate, but the differences were not significant. Administration of fenofibrate lowered serum insulin levels, but not serum glucose levels. As shown in Table 2, the circulating IGF-1 levels were decreased by ~45% in groups 2 and 3 relative to those in group 1 (group 2: *p* < 0.01; group 3: *p* < 0.001).

### 2.2. Discussion

In the current study, we showed that dietary administration of fenofibrate, a PPARα ligand, inhibited 4-NQO-induced pulmonary proliferative lesions (bronchioloalveolar hyperplasia, adenoma, and adenocarcinoma) in male TSOD obese hyperlipidemic mice. Fenofibrate in the diet at a dose of 0.01% (about 9 mg/kg body weight/day) and 0.05% (about 65 mg/body weight/day) are equivalent to approximately 0.3 and 1.5 times the maximum recommended human dose on basis of mg/m^2^ surface area, respectively, and 3 and 20 times the human dose based on body weight comparisons, respectively. These inhibitory effects of fenofibrate seemed to involve modulation of IGF-1R expression. To our knowledge, this is the first report showing that a PPARα agonist could prevent the development of chemically induced pulmonary proliferative lesions in TSOD mice, an animal model of obesity and hyperlipidemia. In this study, a single subcutaneous injection of 4-NQO induced a variety of lung proliferative lesions in male TSOD mice within 30 weeks, although the incidence and number of lung lesions were relatively low.

Li *et al.* [27] reported that PPARα and PPARγ were expressed in the nucleus of NNK-induced lung lesions. They also reported that administration of a synthetic PPARγ ligand significantly inhibited the development of lung lesions induced by NNK. In the current study, we found that PPARα protein expression was upregulated in the nuclei of lung proliferative lesions, including adenoma and adenocarcinoma. However, we did not detect PPARγ protein in these lesions, which may be due to the use of different carcinogens. Interestingly, it was reported that fenofibrate reduced the incidence of oral tumors and suppressed 4-NQO-induced tumor progression in mice [28], suggesting that fenofibrate may be effective for preventing head and neck cancers.

In the current study, the proliferative lesions induced by 4-NQO expressed IGF-1R, p-Akt, and p-Erk. Recently, it was reported that metformin, an antidiabetic agent, inhibited NNK-induced lung tumorigenesis by markedly inhibiting the expression of mammalian target of rapamycin (mTOR) in lung tumors [9]. Metformin also decreased the phosphorylation of IGF-1R/insulin receptor, ERK, and Akt in lung tissues, and thus inhibited the mTOR signaling pathway [9]. Therefore, the insulin–IGF-1 axis may be involved in the development of lung adenocarcinoma, and inhibition of this axis may be beneficial for the suppression of lung proliferative lesions, including adenocarcinoma [11].

In this study, the serum insulin levels in TSOD mice were reduced by fenofibrate. The results of epidemiological studies suggest that non-diabetic patients with elevated serum insulin levels have an increased risk of cancer and cancer-related death [29,30]. The administration of insulin and oral insulin secretagogues was associated with an increased overall risk of cancer, especially lung cancer [31,32]. Regarding the pathogenesis of obesity-related carcinogenesis, the current consensus is that the IGF-1 pathway plays a critical role in insulin-related tumor growth in the lung [33]. Binding of IGF-1 to IGF-1R activates the downstream signal cascade, and triggers cell proliferation in several tissues, including the lung [34]. Furthermore, hyperinsulinemia indirectly increased the bioavailability of IGF-1 by regulating the expression levels of IGF-binding proteins [35]. In a preclinical study of obese Otsuka Long Evans Tokushima Fatty (OLETF) rats, it was found that hyperlipidemia and hyperinsulinemia facilitated carcinogenesis in the colon [36]. Fenofibrate inhibited the development of diabetes in OLETF rats by reducing adiposity, improving peripheral insulin action, and exerting beneficial effects on pancreatic β cells [37]. In the current study, we found that fenofibrate improved the morphology of pancreatic islets (data not shown) and insulin secretion in TSOD mice administered with 4-NQO.

## 3. Experimental Section

### 3.1. Animals, Diet, and Chemicals

Male TSOD mice were obtained at 5 weeks of age from the Institute for Animal Reproduction (Ibaraki, Japan). Three to five mice were housed per cage containing pulp-chip bedding in an air-conditioned animal room at 23 ± 2 °C and 50% ± 10% humidity. Food and tap water were available *ad libitum*. Animal husbandry and experimental protocols were conducted in accordance with the use and care of experimental animals, and were approved by the Committee for Animal Research and Welfare of Gifu University (project code #22-30; approved on 2 December 2010). A standard diet, CE-2 (CLEA Japan, Inc., Tokyo, Japan), was used throughout the study. Fenofibrate and 4-NQO were purchased from Sigma-Aldrich Japan (Tokyo, Japan) and Wako Pure Chemical Industries (Osaka, Japan), respectively.

### 3.2. Experimental Design

After a 1-week quarantine, 99 male TSOD mice (6 weeks old) were divided into four experimental groups and one control group (Figure S1). Mice in groups 1 (*n* = 24), 2 (*n* = 24), and 3 (*n* = 25) were given a single subcutaneous injection of 4-NQO (10 mg/kg body weight) suspended in a mixture of olive oil and cholesterol (20:1). Starting 1 week later, mice in groups 2 and 3 were given experimental diets containing 0.01% or 0.05% fenofibrate, respectively, for a further 29 weeks. Mice in group 4 (*n* = 13) received the diet containing 0.05% fenofibrate for 29 weeks. Group 5 (*n* = 13) served as an untreated control group.

At week 30, all of the animals were euthanized by exsanguination of the abdominal aorta under isoflurane anesthesia, and their lungs, livers, pancreas and kidneys were excised and weighed. The lungs were infused through the bronchi with 10% neutral-buffered formalin and were carefully inspected for the presence of lung tumors using a stereomicroscope (SZX16; Olympus, Tokyo, Japan). After counting the lung tumors, they were excised under the stereomicroscope. All tissues were routinely processed, embedded in paraffin, serially sectioned to 4 μm thick, and stained with hematoxylin and eosin (H&E) for histopathological examination. The lung proliferative lesions were diagnosed based on previously established criteria and were categorized as bronchioloalveolar hyperplasia, adenoma, or adenocarcinoma [38].

Five mice in each group were fasted for 8 h before being sacrificed. At sacrifice, blood was collected from the abdominal aorta and centrifuged at 2000 rpm for 20 min to separate serum. Hematological tests were performed at SRL Inc. (Tokyo, Japan), and included the measurement of total cholesterol (TC), triglyceride (TG), and free fatty acid (FFA) levels. Blood glucose levels were measured using a FreeStyle Freedom (Nipro, Tokyo, Japan).

### 3.3. Immunohistochemistry

Formalin-fixed lung tissues were embedded in paraffin, sectioned, and placed on slides for immunohistochemistry. The primary antibodies were rabbit anti-IGF-1R (Cell Signaling Technology, Inc., Danvers, MA, USA, Catalog #9750, 1:200 dilution), rabbit anti-p-Akt (Ser473, Cell Signaling Technology, Catalog #4060, 1:50 dilution), and rabbit anti-p-p44/42 MAPK (Erk1/2; Thr202/Tyr204, Cell Signaling Technology, Catalog #4370, 1:400 dilution), rabbit anti-PPARα (Santa Cruz Biotechnology, Inc., Santa Cruz, CA, USA, Catalog #sc-9000, 1:50 dilution), mouse anti-PPARγ (Santa Cruz Biotechnology, Catalog #sc-7273, 1:50 dilution), and rat anti-Ki-67 (DAKO, Carpinteria, CA, USA, Catalog #M7249, 1:25 dilution). After deparaffinization and rehydration, all sections were soaked in 3% hydrogen peroxide in methanol for 10 min to remove endogenous peroxidase activity. Antigen retrieval was performed with 0.01 M citrate buffer (pH 6.0) at 121 °C. Sections were then blocked with 2% BSA in PBS to reduce non-specific staining and incubated with primary Abs overnight at 4 °C. Antibodies were detected using VECTASTAIN Elite ABC kits (Vector Laboratories, Burlingame, CA, USA), followed by a chromogenic reaction with 3,3′-diaminobenzidine tetrahydrochloride (DAB; Sigma-Aldrich, St. Louis, MO, USA). The sections were then counterstained with hematoxylin.

The Ki-67 labeling index of lung proliferative lesions was determined using Ki-67-stained immunohistochemical sections for 4–7 mice per group. Ki-67 status was determined in >400 cells per each lesion, and the Ki-67 labeling index was expressed as the percentage of cells stained with the Ki-67 antibody.

### 3.4. Plasma IGF-1 and Insulin Levels

Plasma IGF-1 and insulin levels were measured using a Mouse/Rat IGF-1 Quantikine enzyme-linked immunosorbent assay (R&D Systems, Minneapolis, MN, USA) and a Mouse Insulin enzyme-linked immunosorbent assay (Shibayagi, Gunma, Japan), respectively. IGF-1 and insulin levels were measured in plasma samples obtained from five mice per group at the end of the study.

### 3.5. Statistical Analysis

Data are expressed as the mean ± standard deviation, and the significance of differences among the five groups was determined by one-way analysis of variance with the Tukey–Kramer *post hoc* test. The incidence of pulmonary proliferative lesions was compared among the 5 groups using Fisher’s exact probability test. Differences were considered to be significant at *p* <0.05.

## 4. Conclusions

The results of this study indicate that obese TSOD mice, which exhibited hyperinsulinemia and hyperlipidemia, developed pulmonary proliferative lesions following a single dose of 4-NQO. Dietary administration of fenofibrate effectively inhibited obesity-related lung tumorigenesis by inhibiting the insulin–IGF axis and improving hyperinsulinemia. Our findings suggested that well-tolerated PPARα agonists, such as fenofibrate, may have clinical benefits beyond their established use as lipid-lowering drugs by preventing lung carcinogenesis.

## Figures and Tables

**Table 1.** Incidence and multiplicity (Number of lesions/mouse) of pulmonary proliferative lesions.

Group	Treatment (number of mice examined)	Number of mice with proliferative lung lesions (%)	Number of proliferative lung lesion/mouse			

			Hyperplasia	Adenoma	Adenocarcinoma	Total
1	4-NQO (24)	8/24 (33)	0.21 ± 0.41 a	0.17 ± 0.48	0.08 ± 0.28	0.46 ± 0.78
2	4-NQO—0.01% fenofibrate (24)	4/24 (17)	0.13 ± 0.45	0.08 ± 0.28	0	0.21 ± 0.51
3	4-NQO—0.05% fenofibrate (25)	1/25 (4) b	0	0.04 ± 0.20	0	0.04 ± 0.20 c
4	0.05% fenofibrate (13)	1/13 (8)	0	0.08 ± 0.28	0	0.08 ± 0.28
5	Non-treatment (13)	1/13 (8)	0	0.08 ± 0.28	0	0.08 ± 0.28

a , Mean ± SD;

b , Significantly different from group 1 by Fisher’s exact probability test (*p* < 0.05);

c , Significantly different from group 1 by Tukey-Kramer test (*p* < 0.05).

**Table 2.** Serum levels of triglyceride, free fatty acid, total cholesterol, glucose and insulin of mice at week 30.

Group	Treatment (number of mice examined)	Triglyceride (mg/dL)	Free fatty acid (mEq/L)	Total cholesterol (mg/dL)	Glucose (mg/dL)	Insulin (ng/dL)	IGF-1 (ng/dL)
1	4-NQO (5)	153.6 ± 43.2 a	2235.6 ± 542.5	266.0 ± 48.2	128.4 ± 9.1	7.89 ± 2.71	547.4 ± 58.4
2	4-NQO—0.01% fenofibrate (5)	161.2 ± 17.0	1540.8 ± 365.5 b	250.0 ± 25.0	130.2 ± 24.4	1.31 ± 0.83 c	456.3 ± 15.7 c
3	4-NQO—0.05% fenofibrate (5)	60.8 ± 19.0 c,d	1154.4 ± 80.0 c	201.8 ± 27.4	131.0 ± 11.7	0.16 ± 0.05 c	367.1 ± 16.0 c
4	0.05% fenofibrate (5)	119.8 ± 28.0 e	1218.4 ± 148.1 f	217.4 ± 48.3	109.0 ± 14.7	0.05 ± 0.04 e	317.7 ± 47.8 e
5	Non-treatment (5)	208.2 ± 44.7	1876.8 ± 267.7	238.0 ± 60.4	113.6 ± 16.2	10.09 ± 1.07	573.8 ± 23.6

a , Mean ± SD;

b , Significantly different from group 1 (*p* < 0.05);

c , Significantly different from group 1 (*p* < 0.01);

d , Significantly different from group 2 (*p* < 0.01);

e , Significantly different from group 5 (*p* < 0.01);

f , Significantly different from group 5 (*p* < 0.05).
